# Clinical features and outcomes of COVID-19 and dengue co-infection: a systematic review

**DOI:** 10.1186/s12879-021-06409-9

**Published:** 2021-08-02

**Authors:** Tsheten Tsheten, Archie C. A. Clements, Darren J. Gray, Ripon K. Adhikary, Kinley Wangdi

**Affiliations:** 1 Research School of Population Health, College of Health and Medicine, Australian National University, Canberra, Australia; 2 Royal Centre for Disease Control, Ministry of Health, Thimphu, Bhutan; 3 Telethon Kids Institute, Nedlands, Australia; 4 Curtin University, Perth, Australia

**Keywords:** Dengue, COVID-19, Co-infection, Clinical features, Symptoms, Outcomes

## Abstract

**Background:**

Dengue is the most common arboviral disease in the tropical and sub-tropical regions of the world. Like other regions, dengue-endemic areas have faced the additional public health and socio-economic impact of the ongoing coronavirus disease 2019 (COVID-19) pandemic. COVID-19 and dengue co-infections have been reported, with complicated patient management and care requirements. This review aimed to collate and synthesise current knowledge on the clinical features and outcomes of COVID-19 and dengue virus co-infection, a potentially important new dimension to be considered in public health management of the COVID-19 pandemic.

**Methods:**

A systematic literature review was conducted using PubMed, Web of Science and Scopus databases from 1st January to 21st November 2020. The key search terms used were “dengue” and “coronavirus”. Descriptive analysis with graphical illustrations were used to present the clinical and laboratory parameters of the co-infection.

**Results:**

Thirteen published papers and four news articles were included in the review. Most studies were case reports with a detailed description of the clinical and laboratory characteristics of the co-infection. All cases were in adults with the exception of a six-year old child. The common symptoms of co-infection were fever, dyspnea, headache, and cough. Common laboratory results included thrombocytopenia, lymphocytopenia, elevated transaminases, and leukopenia. Serious outcomes of co-infection included septic shock, acute respiratory disease syndrome and multi-organ failure, leading to death in some patients.

**Conclusions:**

COVID-19 and dengue co-infection was associated with severe disease and fatal outcomes. The correct diagnosis and treatment of co-infection poses a substantial challenge due to the overlapping clinical and laboratory parameters. Therefore, confirmative diagnostic tests are necessary for accurate and timely diagnosis and patient management.

**Supplementary Information:**

The online version contains supplementary material available at 10.1186/s12879-021-06409-9.

## Background

Coronavirus disease 2019 (COVID-19) is a serious respiratory illness caused by severe acute respiratory syndrome coronavirus 2 (SARS-CoV-2) [1]. COVID-19 presents as a respiratory syndrome, mostly characterized by fever and cough [2]. SARS-CoV-2 was first identified from a cluster of patients admitted with pneumonia of unknown etiology to hospitals in Wuhan, Hubei Province, China in December 2019. These patients were epidemiologically linked to a seafood wholesale market where live animals were sold [3]. The number of cases increased across China and eventually spread to the whole world, leading to the declaration of a pandemic. As of June 22, 2021, there were 177,866,160 cases and 3,857,974 deaths globally across 200 countries [4]. During this pandemic, dengue cases increased in many countries. Dengue cases in Brazil have increased by nearly 19% between December 2019 and February 2020 as compared to the same period in 2019 [5]. Thailand reported 61,662 cases in all 77 provinces and 41 deaths in 2020 [6]. Ecuador reported one of the largest dengue outbreaks in its history during this pandemic period [7]. Even places that had never reported dengue in the past saw dengue outbreaks and associated deaths [5].

Dengue is the most common arboviral infection affecting humans in tropical and subtropical regions of the world. Every year, an estimated 96 million dengue infections are reported with 21,000 deaths worldwide [8, 9]. In the last 50 years, the global incidence of dengue has increased 30-fold [10]. Dengue infection presents with a wide range of signs and symptoms including fever, headache, arthromyalgia, retro-orbital pain and rash. Approximately 1 in 20 of those patients develop severe dengue, characterized by plasma leakage, severe bleeding and severe organ impairment [11]. Severe dengue is the leading cause of hospitalization and the number one killer among mosquito-borne diseases in the South-East Asia (SEA) region [12, 13].

The COVID-19 pandemic in dengue-endemic areas is a public health concern because of the overlapping clinical and laboratory features of these diseases. This causes challenges in the correct diagnosis and management of both diseases [3]. Despite similarities in signs and symptoms (like fever, headache and body pain), and laboratory characteristics (like thrombocytopenia and leukopenia) of these two diseases, the management of these diseases are completely different [11, 14]. Hence, specific tests using real-time reverse transcription polymerase chain reaction (RT-PCR) or enzyme-linked immunosorbent assay (ELISA) are necessary to confirm the diagnosis of these diseases. Further, infection with the dengue virus has been reported in SARS-CoV-2 infected patients during the pandemic [15, 16]. Co-infection with these diseases has been associated with higher morbidity than single infections [17, 18]. However, there is a paucity of evidence on the impact of the pathogenesis and prognosis of co-infection between dengue and SARS-CoV-2. This information is crucial for selecting the best course of treatment for the patient and devising appropriate public health policies. Therefore, this review aimed to synthesize the available evidence on the clinical features and outcomes of SARS-CoV-2 and dengue virus co-infection.

## Methods

We conducted a systematic review following the Preferred Reporting Items for Systematic Reviews and Meta-Analyses (PRISMA) guidelines to characterize the clinical, laboratory and disease severity of confirmed SARS-CoV-2 and dengue virus co-infection [19]. Only descriptive analysis was performed as case reports do not have a denominator for any variables to be included in the meta-analysis.

### Search strategy

A systematic search was undertaken in PubMed, Scopus and Web of Science from 1st January 2020 to 21st November 2020. The review focussed on reports of co-infection in patients with dengue and SARS-CoV-2 infection. The search terms included (“dengue”, “coronavirus” and dates (January 2020 ─ November 2020)). Due to the scarcity of published papers, we used these broad search terms to ensnare all published articles reporting SARS-CoV-2 and dengue virus co-infection. We used a broad term as “Coronavirus” to retrieve articles with any kind of Coronavirus species. This was felt important because we came across some articles with different combination of words for the COVID-19 virus. We also pilot-tested and validated our search terms by cross-examining already known articles, i.e., whether our search terms can retrieve already known and eligible articles. Studies were restricted to publications in the English language only.

Inclusion criteria were: (1) any study designs involving humans; (2) all sex/age categories; (3) laboratory-confirmed COVID-19 and dengue. Exclusion criteria were: (1) systematic and narrative reviews, conference abstracts, protocols and perspectives; (2) studies in animals; (3) in-vitro studies; (4) studies without laboratory diagnosis; (5) false-positive laboratory tests and (6) articles in languages other than English. There was no restriction to the study location.

### Selection of studies

All retrieved articles were imported into the free online Rayyan platform for reference management (http://rayyan.qcri.org/) [20]. Two researchers (TT and RKA) independently screened the titles and abstracts on the Rayyan platform. The selected studies underwent full-text articles screening by the same authors. Any discrepancies during this process were resolved by discussion/consultation with the third party (KW). All further library management including citations was conducted using EndNote X7.7.1 (Clarivate Analytics).

### Data extraction and analysis

A Microsoft Excel Worksheet (Microsoft Cooperation) was used to extract data from the included studies. Data extraction was done independently by two authors (TT and RKA) using the same data extraction template. The data were compared and the differences were resolved by consensus. The extracted information included: name of the first author, year of publication, location (country) and setting (emergency, intensive care unit [ICU]), study size, methods of detection of co-infection, age, sex, and outcomes including symptoms and prognosis.

The primary outcome was the clinical and laboratory characteristics of confirmed SARS-CoV-2 and dengue virus co-infection.

Quality scoring was not conducted and no protocol was registered for this systematic review.

## Results

### Study selection

**Fig. 1:**
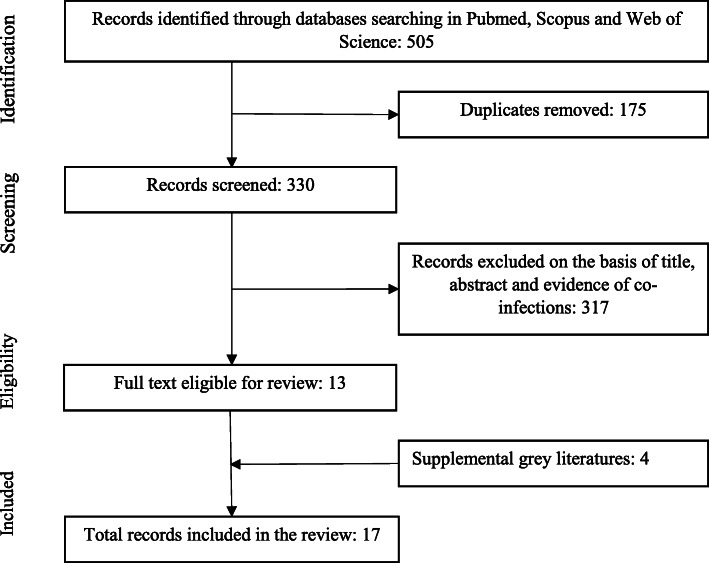
Flow diagram of the study selection process

The search in PubMed, Scopus and Web of Science yielded a total of 505 citations. After removing the duplicates, 330 articles were screened for titles, abstract and co-infections. Three hundred and seventeen articles did not meet the eligibility criteria. The full text of the remaining 13 articles was reviewed. Four news articles obtained via a search of the grey literature that reported SARS-CoV-2 and dengue virus co-infection were also included in this review (Fig. 1).

### Study characteristics

Most of the studies (*n* = 12) were case reports with in-depth description of the clinical and health outcome [15–18, 21–27]. These studies also described the laboratory and treatment outcomes of the co-infection. The four reports from the grey literature presented only the case numbers without clinical and laboratory details [28–31]. Co-infections were reported in eight countries: Argentina (*n* = 1) [25], Brazil (*n* = 3) [18, 21, 22], France (*n* = 2) [15, 16], India (*n* = 1) [32], Indonesia (*n* = 4) [17, 23, 26, 33], Pakistan (*n* = 1) [27] and Thailand (*n* = 1) [24]. The four grey literature articles were from Bangladesh (*n* = 2) [28, 29] and India (*n* = 2) [30, 31] (Table 1). Indonesia reported the most cases of co-infection followed by Pakistan and India. Most co-infection was in adults [15–18, 21, 22, 24–27, 32] and only one study included a paediatric patient [23]. In terms of diagnosis, rapid diagnostic test (RDT) (*n* = 8) [15, 17, 22, 23, 25, 26, 32, 33], ELISA (*n* = 3) [18, 24, 33] and RT-PCR (*n* = 5) [15, 16, 21, 22, 27] were used for the diagnosis of dengue, while RDT (*n* = 3) [17, 21, 23] and RT-PCR (*n* = 11) [15, 16, 18, 21, 22, 24–27, 32, 33] were used for COVID-19 (Table 1).

**Table 1:** Characteristics of studies included in the review of SARS-CoV-2 and dengue virus co-infection

Author (Reference)	Month/Year	Country	Case (n)	Setting	Test method
Pontes RL (22)	May 2020	Brazil	1	Emergency	COVID-19: RDT & RT-PCR; Dengue: RT-PCR
Verduyn et al. (16)	April 2020	France	1	Emergency	COVID-19: RT-PCR; Dengue: RDT & RT-PCR
Bicudo et al. (23)	April 2020	Brazil	1	–	COVID-19: RT-PCR; Dengue: RDT & RT-PCR
Epelboin et al. (17)	March 2020	France	1	–	COVID-19: RT-PCR; Dengue: RT-PCR
Kembuan GJ (18)		Indonesia	1	Ambulatory & ICU	COVID-19: RDT; Dengue: RDT
Somasetia et al. (24)	June 2020	Indonesia	1	Ambulatory & Emergency	COVID-19: RDT; Dengue: RDT
Estofolete et al. (19)	May 2020	Brazil	1	Ambulatory, Emergency & ICU	COVID-19: RT-PCR; Dengue: ELISA
Ratnarathon et al. (25)	January 2020	Thailand	1	Ambulatory & inpatient	COVID-19: RT-PCR; Dengue: RDT
Radisic et al. (26)	April 2020	Argentina	1	Inpatient	COVID-19: RT-PCR; Dengue: RDT
Masyeni et al. (27)		Indonesia	1	Inpatient	COVID-19: RT-PCR; Dengue: RDT
Saddique et al. (28)	June, 2020	Pakistan	5	ICU	COVID-19: RT-PCR; Dengue: RT-PCR
Lokida et al. (34)	Not mentioned	Indonesia	7	Inpatient	COVID-19: RT-PCR; Dengue: RDT, ELISA &, RT-PCR
Mahajan et al. (33)	Not mentioned	India	1	Inpatient	COVID-19: RT-PCR; Dengue: RDT
The Times of India (32)	April 2020	India	1	–	–
The Daily Star (30)	May 23, 2020	Bangladesh	1	–	–
The Daily Star (29)	May 15, 2020	Bangladesh	1	–	–
The Print (31)	August 8, 2020	India	1	–	–

RT-PCR: Reverse transcription polymerase chain reaction; RDT: Rapid diagnostic test; ELISA: Enzyme-linked immunosorbent assay; ICU: Intensive care unit

### Demography and comorbidities

Most co-infections were reported in adults aged 18–69 years [15–18, 21, 22, 24–27, 32]. The youngest patient was a six-year old child [23]. Twice as many males as females were reported to be co-infected (Male:Female: 2:1). Diabetes, cardiovascular disease, hypertension and digestive system disorder were co-morbidities reported in four patients, all adults [17, 18, 27] (Table 2).

**Table 2:** Demography and co-morbidities of SARS-CoV-2 and dengue virus co-infection

Source (author)	Age (years)	Sex	Co-morbidities			
			Diabetes	Cardiovascular	Hypertension	Digestive
Pontes RL (22)	39	M	0	0	0	0
Verduyn et al. (16)	18	M	0	0	0	0
Bicudo et al. (23)	56	F	0	0	0	0
Epelboin et al. (17)	44	M	0	0	0	0
Kembuan GJ (18)	53	M	1	0	0	0
Somasetia et al. (24)	6	M	0	0	0	0
Estofolete et al. (19)	60	F	0	0	1	0
Ratnarathon et al. (25)	35	M	0	0	0	0
Radisic et al. (26)	25	M	0	0	0	0
Masyeni et al. (27)	69	F	0	0	0	0
Saddique et al. (28)	43 (25–50)	M: 3 F: 2	2	2	0	1
Lokida et al. (34)	NA	NA	NA	NA	NA	NA
Mahajan et al. (33)	22	F	0	0	0	0
The Times of India (32)	68	M	NA	NA	NA	NA
The Daily Star (30)	NA	NA	NA	NA	NA	NA
The Daily Star (29)	53	M	NA	NA	NA	NA
The Print (31)	NA	NA	NA	NA	NA	NA
Total	–	–	3	2	1	1

F: Female; M: Male; NA: Not applicable/Not reported

### Clinical and laboratory characteristics

**Fig. 2:**
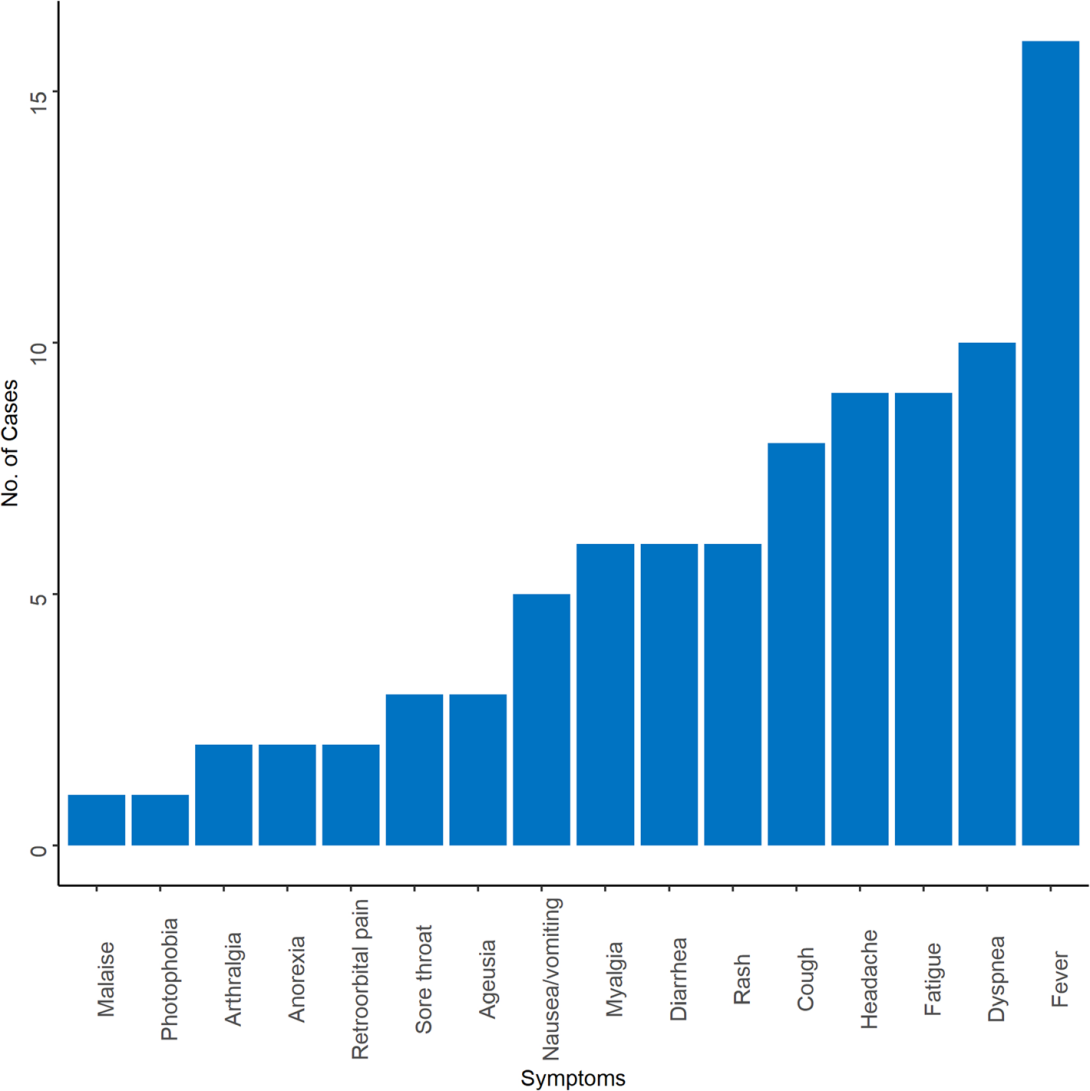
Characteristics of symptoms of SARS-CoV-2 and dengue virus co-infection

The clinical records of 16 cases were extracted. Fever was present in all 16 patients [15–18, 21–27, 32]. Other clinical manifestations were dyspnea (*n* = 10) [15, 17, 18, 21–24, 27], fatigue (*n* = 7) [15, 16, 23, 25, 27], headache (*n* = 9) [15, 16, 18, 22, 25, 27] and cough (*n* = 8) [15, 18, 22, 24, 26, 27]. Patients also presented arthralgia (*n* = 2) [25, 26], anorexia (*n* = 2) [15, 16], retro-orbital pain (*n* = 2) [15, 18], malaise (*n* = 1) [17] and photophobia (*n* = 1) [15] (Fig. 2).

**Fig. 3:**
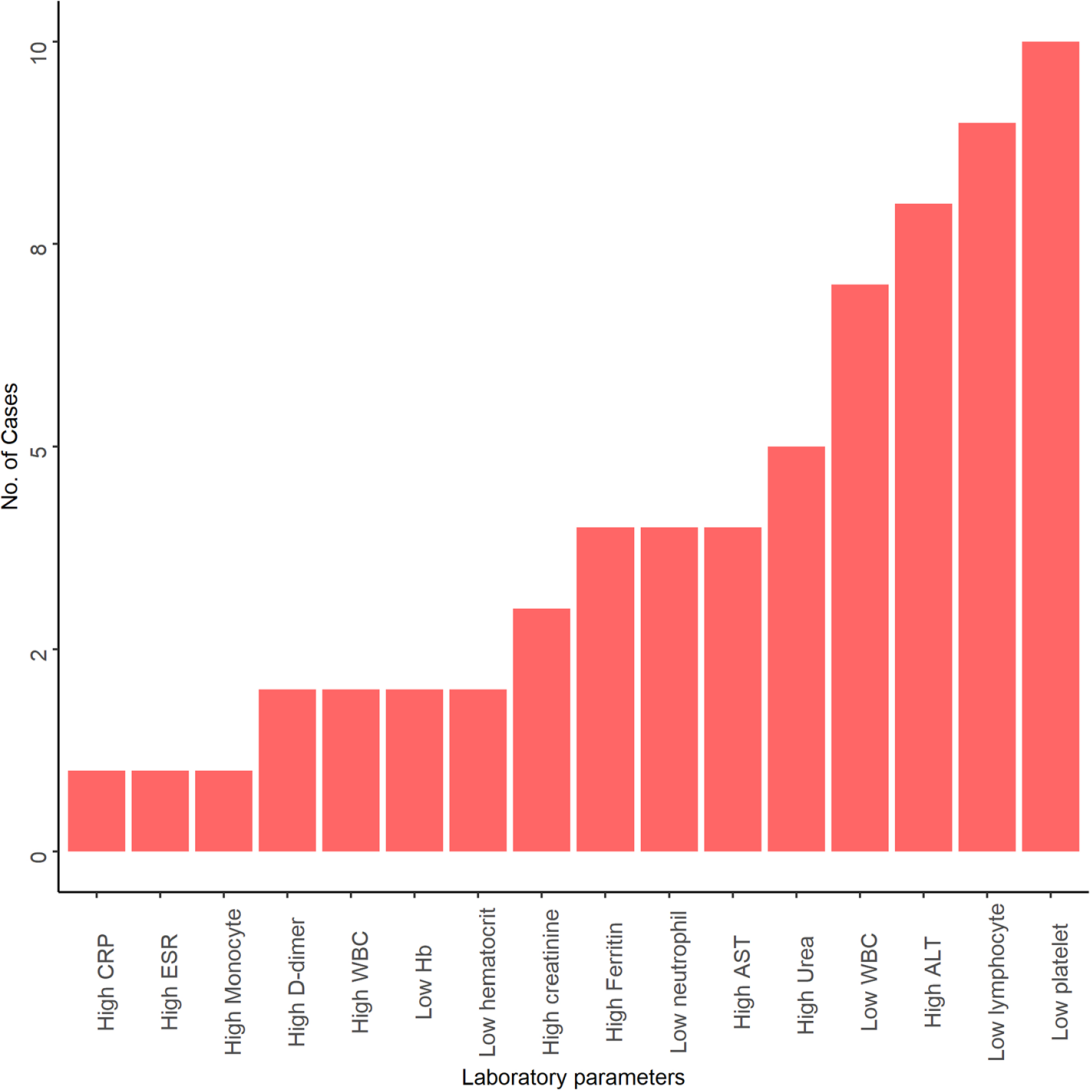
Characteristics of laboratory findings of SARS-CoV-2 and dengue virus co-infection

The most frequently reported laboratory findings were thrombocytopenia (*n* = 10) [15, 17, 18, 22, 24, 27], lymphopenia (*n* = 9) [15, 17, 22, 24, 25, 27], elevated alanine aminotransferase (*n* = 8) [18, 22–24, 27] and leukopenia (*n* = 7) [15, 17, 22, 26, 27]. Other less commonly reported laboratory findings were elevated D-dimer (*n* = 2) [22, 25], leucocytosis (*n* = 2) [23, 32], reduced haemoglobin (*n* = 2) [23, 27], reduced haematocrit (*n* = 2) [23, 24], high C-reactive protein (*n* = 1) [22], monocytosis (*n* = 1) [26] and high erythrocyte sedimentation rate (*n* = 1) [26] (Fig. 3). The detailed description of clinical and laboratory characteristics of each case is highlighted in the Additional File 1.

Chest X-ray (CXR) or computed tomography (CT) images with ground-glass opacity in the lungs were reported in five patients [17, 18, 22, 24, 25], while the remaining patients had a normal CXR or CT finding [15, 23, 26, 32]. Bilateral lung opacities were presented in four patients [17, 18, 22, 24], but one patient presented with a right lower lobe infiltrate and mild splenomegaly [25].

### Clinical outcomes

Three patients presented complications related to shock, acute respiratory distress syndrome (ARDS) and multiple organ failure. Six had fatal outcomes [17, 18, 23]. The median days to recovery from co-infection was 11 and ranged from 9 to 20 days.

## Discussion

Knowledge of the clinical and laboratory features of SARS-CoV-2 and dengue virus co-infection is critical for correct diagnosis and patient management. This systematic review was conducted using available information from case reports or case series in absence of large population-based studies. Whilst numbers were small, co-infection was predominantly in the SEA region and reported in adults. Fever was the most commonly reported symptom, while thrombocytopenia was the most common laboratory finding. Co-infections were often severe in terms of morbidity and had a high risk of death, associated with complications such as septic shock, acute respiratory disease syndrome and multi-organ failure.

Dengue continues to be an important disease in the SEA region, with the circulation of multiple dengue virus serotypes [34]. Dengue emerged in the region after World War II as a result of the introduction of the principal dengue vector, *Aedes aegypti*, due to trade and the movement of people [35, 36]. The region now bears a high burden of dengue with 1.8 billion people living at risk of dengue infection [37]. In addition, all countries in the region also faced the deadly rampage of COVID-19, causing considerable disease burden and socio-economic disruptions [38–42]. Thailand was the first country outside China to identify SARS-CoV-2, probably because it is a popular destination for international tourists, especially from China [39]. Additionally, many people move across the land border into Thailand every day, posing an immediate threat to the local population through transmission of COVID-19 [43]. In Bhutan and Indonesia, there are mounting reports of cases associated with the non-stop repatriation of migrant workers from the worst affected countries [42, 44]. India was one of the most hard hit countries by COVID-19 globally and ranks second in terms of cumulative cases and third in terms of cumulative deaths [45]. Widespread distribution of both cases and deaths are now evident in the region [4, 44, 46]. Countries in Latin America, such as Brazil, have witnessed extensive dengue outbreaks over the last three decades. Brazil contributes around 55% of the disease burden in the American continent [47], and is also one of the countries worst affected by the COVID-19 pandemic. Globally, the Americas had the highest cumulative cases and deaths attributable to COVID-19 [4]. Latin America is likely to be one regions of the world where dengue and SARS-CoV-2 co-infection presents an important threat to public health.

Adult males predominated among co-infected individuals, with only one case of co-infection in a child reported [23]. Adults and males were found to have a higher incidence of SARS-CoV-2 than children and females in a nation-wide study conducted in mainland China [48]. This was attributed to greater community contact, including increased outdoor activities, visiting shopping centres, dining in restaurants and bars, and gathering in colleges and universities [49–51]. Similarly, there is a wealth of evidence on the increase of dengue incidence among these economically productive age groups, reported in almost all the tropical and sub-tropical regions of the world [52, 53]. Hence, mitigation efforts and preventive measures targeting adults might be more important for SARS-CoV-2 and dengue virus co-infection.

Fever was the most common symptom manifested by all co-infected patients. Fever is the most common symptom for both dengue and COVID-19, which poses a challenge in making a correct diagnosis for either infection at the current time [3, 11, 32]. However, the presentation of additional symptoms such as dyspnea (breathing problems), cough, headache and ageusia, among other clinical features are more likely to guide clinicians to suspect COVID-19 and eventually confirm it using SARS-CoV-2 tests [17, 18, 22]. These symptoms were consistently found in other studies including a meta-analysis of symptoms among COVID-19 patients [54].

In the current review, COVID-19 was not usually included as a differential diagnosis in dengue patients without evidence of breathing problems or cough. COVID-19 tests were performed and confirmed when there was no improvement following treatment for dengue [21]. In one study, a 35-year old man was diagnosed with dengue and was tested for COVID-19 when the patient’s health deteriorated, as indicated by the development of bilateral alveolar infiltration in the lung about one week after the onset of symptoms. The delayed diagnosis of COVID-19 and non-compliance with COVID-19 prevention protocols led to hospital-acquired SARS-CoV-2 infection in a healthcare provider [24]. The non-specific clinical symptoms of COVID-19 and dengue calls for including both the diseases in the differential diagnosis of acute febrile illness patients at the current time [42].

In this review, thrombocytopenia was the predominant laboratory finding of SARS-CoV-2 and dengue virus co-infection. Both diseases seem to follow similar pathophysiological pathways. Thrombocytopenia in these diseases results from depressed platelet synthesis due to virus-induced bone marrow suppression and immune-mediated clearance of platelets [55, 56]. Further, autoantibodies and immune complexes produced in response to SARS-CoV-2 and dengue virus infection destroy platelets [56, 57]. A comparison between co-infection and mono-infection of SARS-CoV-2 and dengue virus in terms of both clinical and laboratory parameters are shown in the Additional File 2. Although a marked variation is observed in COVID-19, dengue has the highest prevalence of thrombocytopenia in all the reported studies.

Studies in the past have shown that patients with comorbidities were more likely to result in severe illness and death in both COVID-19 and dengue [54, 58]. Diabetes, hypertension and digestive disease [17, 18, 27] were observed to have significant severe disease outcomes as compared to patients without other chronic diseases [59]. In particular, diabetes and cardiovascular disease were observed to be significant risk factors for severe disease and higher case fatalities [60]. Patients with co-morbidities should take strict precautionary measures to avoid infections with either virus.

This review is subjected to limitations. The findings of this systematic review were based on few studies that were available for inclusion. All of these studies were case reports and there have been no population-based studies reported to date. We also did not include the treatment aspect of the co-infection as it was beyond the scope of this review.

## Conclusions

Co-infection of SARS-CoV-2 and dengue virus is associated with significant morbidity and mortality. Overlapping clinical and laboratory features of each infection poses a challenge in the accurate diagnosis and treatment of cases. Delayed diagnosis of co-infection can result in serious patient complications with poor outcomes. The review underscores the importance of accurate and timely diagnosis using virus-specific tests.

## Supplementary Information

**Additional file 1.** Clinical and laboratory characteristics of SARS-CoV-2 and dengue virus co-infection.

**Additional file 2.** Comparison of the clinical and laboratory characteristic of co-infection, only COVID-19 and only dengue reported in the present and past studies respectively.

**Additional file 3.**

## Data Availability

All data analysed during the current study are available in the Additional File 3.

## Abbreviations

ARDS: Acute Respiratory DistressSyndrome
COVID-19: Coronavirus Disease 2019
CT: Computed tomography scan
CXR: Chest X-ray
ELISA: Enzyme-Linked ImmunosorbentAssay
ICU: Intensive Care Unit
PRISMA: Preferred Reporting Itemsfor Systematic Reviews and Meta-Analyses
RDT: Rapid Diagnostic Test
SARS-CoV-2: Severe acute RespiratorySyndrome Coronavirus 2
SEA: South-East Asia
