# Supplementary material for: Clinical features and outcomes of COVID-19 and dengue co-infection: a systematic review
Source: BMC Infect Dis. 2021 Aug 2;21:729. doi: 10.1186/s12879-021-06409-9 (PMC8327042; doi:10.1186/s12879-021-06409-9)
Supplement: Supplementary file 1 — Additional file 1. Clinical and laboratory characteristics of SARS-CoV-2 and dengue virus co-infection. [file 12879_2021_6409_MOESM1_ESM.docx]

| Author | Clinical characteristics | Laboratory characteristics |
| --- | --- | --- |
| Pontes RL (22) | Fever, myalgia, diarrhoea, ageusia & dyspnea | - |
| Verduyn et al. (16) | Fever, asthenia, anorexia, headache, erythema, arthromyalgia, dyspnea, maculopapular rash, dry cough, retro-orbital pain, photophobia, anorexia, nausea, vomiting & cervical lymphadenopathies | Thrombocytopenia, leukopenia, lymphopenia, neutropenia & subnormal AST/ALT |
| Bicudo et al. (23) | Sore throat, ageusia, headache, fever, dry cough, dyspnea, erythemato-papular rash, diarrhea & nausea | Leukopenia, lymphopenia, thrombocytopenia, elevated D-dimer, elevated CRP, high AST/ALT, high ferritin & atypical lymphocytes |
| Epelboin et al. (17) | Headache, fever, anorexia, fatigue, diarrhoea, ageusia, maculopapular exanthema & positive tourniquet test | Elevated creatinine phosphokinase & AST/ALT |
| Kembuan GJ (18) | Fever, malaise, sore throat, lethargic, dyspnea & positive tourniquet sign | Thrombocytopenia, leukopenia & lymphopenia |
| Somasetia et al. (24) | Fatigue, pale, fever, abdominal pain, altered consciousness & shock | Low haemoglobin, low haematocrit, elevated urea, elevated ferritin, AST/ALT & leucocytosis |
| Estofolete et al. (19) | Fever, myalgia, headache, retro-orbital pain, dry cough & anisocoria | Low platelet, elevated haematocrit, creatinine, D-dimer & AST/ALT |
| Ratnarathon et al. (25) | Fever, myalgia, productive cough, nausea, vomiting & dyspnea | Thrombocytopenia, lymphopenia, reduced haematocrit & elevated ALT |
| Radisic et al. (26) | Asthenia, headache, arthromyalgia, fever & sore throat | Lymphopenia, elevated ferritin & D-dimer |
| Masyeni et al. (27) | Fever, arthromyalgia, nausea & cough | Leukopenia, monocytosis, elevated ESR & hyponatremia |
| Saddique et al. (28) | Fever (5), fatigue (5), rash (3), cough (3), dyspnea (2), headache (4), nausea/vomiting (2) & diarrhea (3) | Leukopenia (3), neutropenia (3), lymphopenia (4), thrombocytopenia (5), elevated ALT (4), alkaline phosphatase (3), urea (4) & creatinine (3) |
| Lokida et al. (34) | - | - |
| Mahajan et al. (33) | Fever | - |
| The Times of India (32) | - | - |
| The Daily Star (30) | - | - |
| The Daily Star (29) | - | - |
| The Print (31) | - | - |
