# Supplementary material for: Clinical features and outcomes of COVID-19 and dengue co-infection: a systematic review
Source: BMC Infect Dis. 2021 Aug 2;21:729. doi: 10.1186/s12879-021-06409-9 (PMC8327042; doi:10.1186/s12879-021-06409-9)
Supplement: Supplementary file 2 — Additional file 2. Comparison of the clinical and laboratory characteristic of co-infection, only COVID-19 and only dengue reported in the present and past studies respectively. [file 12879_2021_6409_MOESM2_ESM.docx]

| Parameters | Current study | COVID-19 | | Dengue | | |
| --- | --- | --- | --- | --- | --- | --- |
|  |  | Rodriguez-Morales AJ et al (1) | [Pormohammad A et al](https://www.ncbi.nlm.nih.gov/pubmed/?term=Pormohammad%20A%5BAuthor%5D&cauthor=true&cauthor_uid=32681968) (2) | [Priyadarshini D et al](https://www.ncbi.nlm.nih.gov/pmc/articles/PMC2806829/) (3) | [Thanachartwet V et al](https://www.ncbi.nlm.nih.gov/pubmed/?term=Thanachartwet%20V%5BAuthor%5D&cauthor=true&cauthor_uid=26468084) (4) | Jain S et al (5) |
| **Clinical** | Number (n) | Prevalence (%) | Prevalence (%) | Prevalence (%) | Prevalence (%) | Prevalence (%) |
| Fever | 16 | 89 | 87 | 100 | 66 | 99 |
| Dyspnea | 10 | 46 | 24 | - | - | 8 |
| Headache | 9 | 8 | 14 | 61 | 86 | - |
| Cough | 8 | 58 | 68 | - | - | - |
| Fatigue | 7 |  | 39 | - | 78 | - |
| Myalgia | 6 | 29 | 24 | 61 | 92 | - |
| Diarrhoea | 6 | 6 | 8 | - | 32 | 10 |
| Rash | 6 | - | - | - | 46 | 18 |
| Nausea/vomiting | 5 | - | 7 | 61 | 16 | 52 |
| Sore throat | 3 | 11 | 14 | - | - | 5 |
| **Laboratory** |  |  |  |  |  |  |
| Low platelet | 10 | - | 13 | - | 62 | 85 |
| Low lymphocyte | 9 | 43 | 58 | - | - |  |
| High ALT | 8 | 24 | - | 79 |  | 19 |
| Low WBC | 7 | 19 | 28 | 50 | - | 34 |
| High AST | 4 | 33 | - | 97 | - | 23 |
| High creatinine | 3 | 21 | - | - | - | - |
| High WBC | 2 | 17 | - | - | - | - |
| High CRP | 1 | 58 | 79 | - | - | - |
| High ESR | 1 | 42 | - | - | - | - |
|  |  |  |  |  |  |  |
| **Outcome** |  |  |  |  |  |  |
| Death | 6 (22%) | 14 | 6 | - | - | - |

**References**

1. Rodriguez-Morales AJ, Cardona-Ospina JA, Gutiérrez-Ocampo E, Villamizar-Peña R, Holguin-Rivera Y, Escalera-Antezana JP, et al. Clinical, laboratory and imaging features of COVID-19: A systematic review and meta-analysis. Travel Med Infect Dis. 2020;34:101623.

2. Pormohammad A, Ghorbani S, Baradaran B, Khatami A, R JT, Mansournia MA, et al. Clinical characteristics, laboratory findings, radiographic signs and outcomes of 61,742 patients with confirmed COVID-19 infection: A systematic review and meta-analysis. Microb Pathog. 2020;147:104390.

3. Priyadarshini D, Gadia RR, Tripathy A, Gurukumar KR, Bhagat A, Patwardhan S, et al. Clinical findings and pro-inflammatory cytokines in dengue patients in Western India: a facility-based study. PLoS One. 2010;5(1):e8709.

4. Thanachartwet V, Oer-Areemitr N, Chamnanchanunt S, Sahassananda D, Jittmittraphap A, Suwannakudt P, et al. Identification of clinical factors associated with severe dengue among Thai adults: a prospective study. BMC Infect Dis. 2015;15:420.

5. Jain S, Mittal A, Sharma SK, Upadhyay AD, Pandey RM, Sinha S, et al. Predictors of Dengue-Related Mortality and Disease Severity in a Tertiary Care Center in North India. Open Forum Infect Dis. 2017;4(2):ofx056.
